# How do anxiety and stress affect soccer referees? An ERPs study

**DOI:** 10.3389/fpsyg.2024.1294864

**Published:** 2024-06-27

**Authors:** Li Zhang, Haonan Shi, Hongfei Zhang, Jianlan Ding, Zilong Wang

**Affiliations:** ^1^School of Marxism, Xi’an Jiaotong University, Xi’an, China; ^2^School of Sports Economics and Sports Management, Xi’an Physical Education University, Xi’an, China; ^3^Graduate Department, Xi’an Physical Education University, Xi’an, China; ^4^Information Technology Department, Shaanxi Police College, Xi’an, China; ^5^Center for Sports Neuroscience Management and Social Behavior Decision-Making, Xi’an Physical Education University, Xi’an, Shaanxi, China

**Keywords:** decision-making, anxiety, pressure, P300, N400

## Abstract

The decision-making of soccer referees is one of the typical forms influenced by factors such as environmental pressure and individual emotions. While previous studies have explored how common factors like personal anxiety and on-field pressure affect the decisions of soccer referees, the mechanisms by which anxiety influences decision-making under pressure remain unclear. This study developed a penalty task based on real soccer match scenarios and recruited 76 experienced soccer referees. These referees were divided into two groups, high anxiety and low anxiety, based on their anxiety levels, to perform decision-making tasks under different pressure environments simulated to mimic real matches. Additionally, this research employed Event-Related Potential (ERP) technology to compare the brain signals of soccer referees with different levels of anxiety when facing foul play under various pressure environments. It was found that referees with high levels of anxiety displayed larger P300 and N400 amplitudes in a low-pressure environment (*p* = 0.0059, *t* = 2.9437). However, no significant differences in P300 and N400 amplitudes were observed between referees with high and low levels of anxiety under high-pressure conditions (*p* = 0.1890, *t* = 1.3411). This study not only reveals the complex mechanisms of anxiety in the decision-making process of referees but also emphasizes the importance of understanding and managing the psychological state of referees in competitive sports to improve the quality of their decisions. Our findings provide an empirical basis for future efforts to mitigate the impact of anxiety and optimize the decision-making process in similar high-pressure environments.

## 1 Introduction

In recent years, the group of football referees has garnered escalating attention across diverse sectors of society, coinciding with the burgeoning impact of football (Plessner and Haar, 2006). As pointed out by Johansen, at present, football referees, much like football stars such as Messi and Ronaldo, face unparalleled scrutiny from fans and media (Johansen and Haugen, 2013). The crucial decisions made by referees in a match can directly impact the ultimate result of the football game, potentially reshaping a team’s destiny—be it victory leading to championship, honor, and substantial rewards, or failure resulting in relegation to a lower-tier league. As a result, ensuring that football referees remain focused and impartial in their judgments during games has emerged as a prominent subject in sports research. Against this backdrop, re-searchers have extensively investigated the factors that could impact football referees’ decision-making (Aragãoe Pina et al., 2018). Existing research indicates that factors influencing referees’ decision-making include on-site crowd pressure (Nevill et al., 1999), referees’ mastery and application of learned judgment skills, pressure from coaches and players (Sutter and Kocher, 2004), unfamiliar refereeing environments, players’ nationalities, attention bias, and referees’ emotional state at the time (Dawson and Dobson, 2010; Di Corrado et al., 2011). Among these factors, the individual emotional factor of referees, particularly anxiety, is regarded as one of the most pivotal factors influencing their judgment decisions (Eysenck and Calvo, 1992; Sors et al., 2019), while the pervasive pressure on the football field further complicates the influence of anxiety on their decisions.

Existing research generally acknowledges anxiety as a common emotion in human decision-making. It is a negative emotional state characterized by tension, worry, and distress, along with activation of the autonomic nervous system to cope with potential threats (Mellalieu et al., 2009). As a prevalent negative emotion (with approximately 25% of individuals experiencing clinically significant levels of anxiety at some point in their lives), anxiety significantly impacts individual decision-making (Mullen et al., 2005; Eysenck et al., 2007; Derakshan and Eysenck, 2009). Researchers posit that this impact may be attributed to the potential inhibition of the prefrontal cortex during anxiety. Given the crucial role of the prefrontal cortex in cognitive control, decision-making, and behavioral regulation, anxiety-induced impairment of prefrontal cortex function can affect individuals’ decision-making abilities and behavioral regulation. Additionally, in certain specific situations, anxiety augments the output of the amygdala-centered pre-attention threat assessment mechanism, directing attention toward threat-related stimuli and affecting task processing efficiency (Mesagno and Mullane-Grant, 2010; Sun and Zhang, 2013).

Football referees, as pointed out by some researchers, are among the professions facing the highest pressure in decision-making (Beilock and Carr, 2005). This phenomenon, which has been present since the first appearance of football referees in 1868, has intensified in recent years (Alonso-Arbiol et al., 2008). Referees, whether officiating in prestigious competitions such as the European Champions League or in leagues with less visibility like those in Norway, universally experience pressure from spectators, coaches, and players, which they perceive as potentially influencing their decision-making and overall performance (Jiménez Sánchez and Lavín, 2020). The sources of these pressures primarily include the fear of failure and making mistakes, time constraints for decision-making during live events, fear of injury, verbal attacks, and most importantly, the noise from the live audience (Otten, 2009). Therefore, what are the impacts of these common pressures on football referees under anxiety? According to some researchers, moderate pressure can alleviate the impact of anxiety on individuals, enabling them to perform better under pressure (Dodson, 1915). Many athletes and referees believe that, when experiencing anxiety, suitable pressure in real games assists them in better concentration and reaching a state of “total absorption and confidence in handling the task at hand,” known as the flow state, leading to improved results and performance. This phenomenon aligns with the “Yerkes-Dodson law” or “Yerkes-Dodson curve,” which describes the relationship between pressure or motivation levels and cognitive or behavioral performance, suggesting that moderate pressure or motivation can enhance performance, while excessive or insufficient pressure or motivation will result in performance decline (Easterbrook, 1959; Diamond et al., 2007). Neuroscientific studies indicate that moderate pressure or motivation can enhance the activity of the neural regulatory system, leading to the release of neurotransmitters and stress hormones, such as adrenaline and cortisol. According to the Yerkes-Dodson law, while anxiety may impair the performance of athletes or referees, moderate pressure enhances the activity of the individual’s neural regulatory system, leading to the release of neurotransmitters and stress hormones, promoting the function of the prefrontal cortex and hippocampus, enhancing performance, and to some extent, counteracting the negative impact of anxiety (Sapolsky, 2015).

Up to now, researchers have obtained initial insights into the effects of anxiety and stress on individual performance in sports activities. However, most of these studies have approached the topic from the perspective of behavioral science, primarily focusing on athletes, while research on groups requiring numerous real-time decisions, like soccer referees, remains limited. In particular, there is a dearth of research on the brain activity patterns and functions in individuals engaged in sports officiating tasks. With the continuous advancements in neuroscience, researchers have extended their investigations in decision science to the neural level (Fu et al., 2022), leveraging sophisticated neuroscientific techniques such as EEG (Electroencephalography) and fMRI (Functional Magnetic Resonance Imaging). Substantial research has unveiled that individual decision-making processes primarily encompass information processing and knowledge extraction. Information processing entails perceiving, encoding, storing, and retrieving external and internal information, alongside processing and integrating this information. Knowledge extraction, on the other hand, involves extracting pertinent knowledge from existing databases, experiences, and learning, and applying it to the decision-making process (Gläscher et al., 2010; Voon et al., 2014; Lebreton et al., 2015). Among all the ERP components, P300 stands out as a well-represented component, reflecting the allocation of decision-making resources during individual cognitive decision-making processes. The amplitude of P300 is closely associated with the decision-making resources utilized by the participant. The higher the number of decision-making resources consumed during the decision-making process, the greater the amplitude of P300 is triggered. Conversely, a lower amplitude of P300 is observed when fewer decision-making resources are consumed (Verleger, 1988; Kok, 2001; Polich, 2007). Similarly, The N400 is a negative waveform that emerges approximately 400 milliseconds after a stimulus is presented, primarily associated with language processing and semantic understanding (Kutas and Federmeier, 2011). Recent research has expanded our understanding of the N400, indicating that its amplitude not only reflects the processing of linguistic materials but is also related to a broader process of knowledge extraction. Specifically, the greater the cognitive load or information processing demands required by subjects to retrieve knowledge related to a stimulus, the larger the amplitude of the N400; conversely, the amplitude decreases when these demands are lesser. P300 and N400 have been widely used in diverse studies investigating functional impairments in individual decision-making, including research on schizophrenia and depression (Laurent et al., 2010). In this context, these components can be employed in studies focusing on the decision-making function of soccer referees. The decision-making processes of soccer referees also entail information processing and knowledge extraction. During matches, referees must rapidly and accurately process information from various sources, such as player actions, ball position, and match time. Simultaneously, referees need to extract and apply the knowledge they have gained from soccer rules and referee training (e.g., judgment rules for fouls, offside, handball, etc.) to enhance their understanding of match events and make suitable judgment decisions. By observing variations in P300 and N400 during decision-making tasks conducted by soccer referees under diverse levels of anxiety and pressure in an experimental setup mimicking real soccer match pressure, we can gain deeper insights into how anxiety and stress affect referees’ decision-making processes. To achieve this goal, the present study has devised a soccer referee judgment decision-making task experiment, intending to investigate the underlying mechanism of how pressure influences judgment decisions in soccer referees with different anxiety levels, while gathering physiological evidence through behavioral data and EEG data.

Drawing from the studies mentioned above, this study formulates the subsequent hypotheses: (1) Anxiety emotions primarily affect the cognitive processing underlying soccer referees’ penalty decisions. Specifically, variances in anxiety levels among referees will yield notable disparities in P300 and N400 amplitudes during decision-making tasks. (2) In line with the “Yerkes-Dodson Law,” the impact of anxiety emotions on the decision-making cognitive processing of soccer referees is expected to diminish under suitable pressure conditions. This will manifest as a significant decrease in the amplitude differences of P300 and N400 elicited by referees with varying levels of anxiety in pressured contexts.

## 2 Materials and methods

### 2.1 Participants

**Figure 1:**
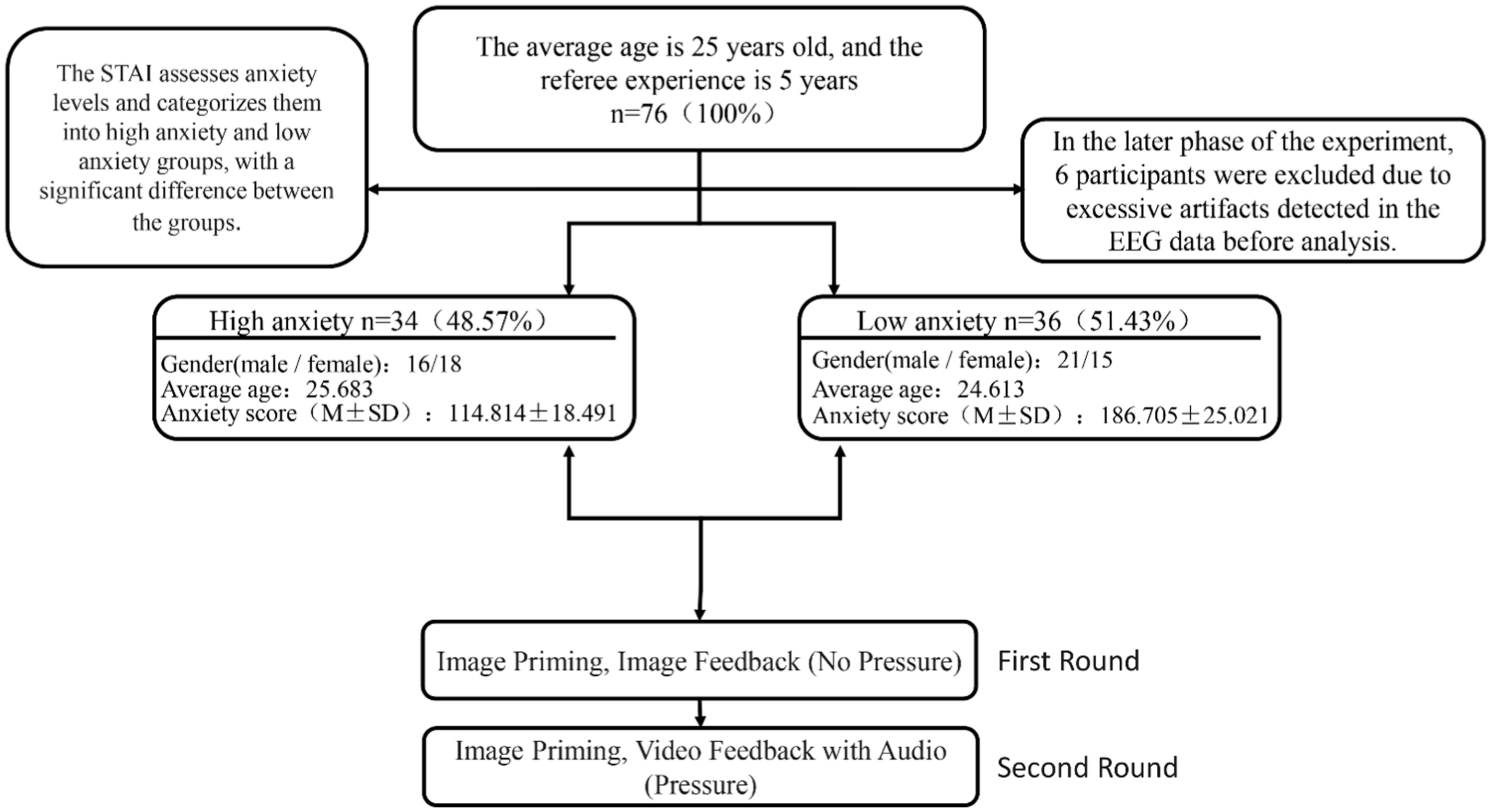
Participants grouping.

This study employed G*Power 3.1.9.4 software to calculate the sample size, considering the effect size from existing research and comprehensive literature discussion, determining a preset medium effect size of 0.15, with α set between 0.05 and 0.80. Although the calculation indicated that data from 45 participants would be sufficient to achieve the expected statistical power, recruiting 76 football referees with significant refereeing experience was deemed necessary to ensure the robustness of the experimental results and to mitigate risks associated with data loss or participant dropout. These 76 referees, with an average age of 25, all underwent professional football referee training during their university years, and have an average refereeing experience of 5 years. Throughout participant recruitment, this study followed criteria encompassing referee experience, professional rank, and diversity. A cohort of 76 football referees, each with over 200 instances of officiating, and holding qualifications issued by the Chinese Football Association, was recruited. This pool encompassed three categories based on football match and referee level: the National University Football League, China League Two, and China League One. Furthermore, the State–Trait Anxiety Inventory (STAI) was utilized to evaluate participants’ anxiety levels and to categorize them (Bieling et al., 1998). Participants were classified into high and low anxiety groups based on their scale scores. Importantly, a substantial discrepancy in anxiety levels was observed between these two groups. In the later phase of the experiment, 6 participants were excluded due to excessive artifacts detected in the EEG data before analysis. As a result, the ultimate division included 34 participants in the high anxiety group and 36 participants in the low anxiety group, as outlined in Figure 1. In line with established standards and norms for behavioral and ERP experiments, participants were mandated to possess uncorrected or corrected normal vision, right-handedness, and a stable mental state. The specific grouping is shown in Figure 1. All participants were compensated for their participation in the experiment and provided informed consent.

### 2.2 Experimental design

The experiment utilized the E-Prime 2.0 system (Psychology Software Tools, Pitts-burgh, PA, United States) for its presentation. Participants were instructed to keep a 70 cm distance between their heads and the screen during the experiment. The screen had a horizontal visual angle of 2.58° and a vertical visual angle of 2.4°. Images were displayed at a resolution of 200 × 150 pixels, ensuring consistent brightness and contrast. Experiment stimuli comprised frames extracted from soccer foul videos during matches. Soccer foul videos for this study were chosen from the “Obvious Foul Group” instructional videos of the International Federation of Association Football (FIFA) 2019 referee training course. Soccer foul videos for this study were chosen from the “Obvious Foul Group” instructional videos of the International Federation of Association Football (FIFA) 2019 referee training course. The experiment occurred in a standard ERP laboratory with gentle indoor lighting and consistent temperature. Participants were directed to adopt their most comfortable sitting posture, release tension in their head and facial muscles, and regulate blinking frequency throughout the experiment. They were required to concentrate their attention and initiate the experiment upon achieving stable brainwave patterns.

As this study aims to investigate how pressure conditions affect soccer referees with varying anxiety levels in actual match scenarios, accurately defining and reproducing real match pressure conditions becomes a pivotal element of the experimental design. Previous research generally indicates that soccer referees typically encounter moderate pressure during most matches (Jordet et al., 2007). Therefore, the study’s researchers posit that moderate pressure more effectively emulates the atmosphere of actual soccer matches. Nevertheless, there currently lacks a standardized academic criterion to define the concept of moderate pressure. This variance stems from discrepancies in how pressure is defined across various research domains and experimental endeavors. Broadly speaking, moderate pressure entails creating a level of psychological stress or challenge in participants that does not lead to undue distress or severe discomfort (Colwell, 2000). Therefore, this study draws upon previous research to define and operationalize pressure levels, as well as to align with the academic community’s agreement on suitable pressure standards. The decision was to replicate the pressure source by utilizing the crowd noise during referees’ penalty judgments (Nevill et al., 2002). After evaluating pertinent research in this domain, the study categorizes pressure and no-pressure settings according to distinct feedback materials. In the no-pressure scenario, feedback materials entail silent images, whereas in the pressure context, feedback materials involve audiovisual snippets of soccer match spectators. Referees perceive either jeers or cheers based on decision outcomes, akin to real soccer match circumstances (Lavie et al., 2004). Drawing on previous research findings, this study maintains the noise level of the audio-visual feedback materials presenting soccer match audience scenes at around 60 decibels (Hardy and Hutchinson, 2007; Hughes et al., 2013). If participants first experience a stressful environment, it may lead to an accumulation of psychological stress. Meanwhile, randomization or balanced design may introduce more data variation in some cases, thereby affecting the final experimental results. To avoid the above situations, this study has all participants first experience low-stress conditions (Baumeister, 1991; Hockey, 1997).

**Figure 2:**
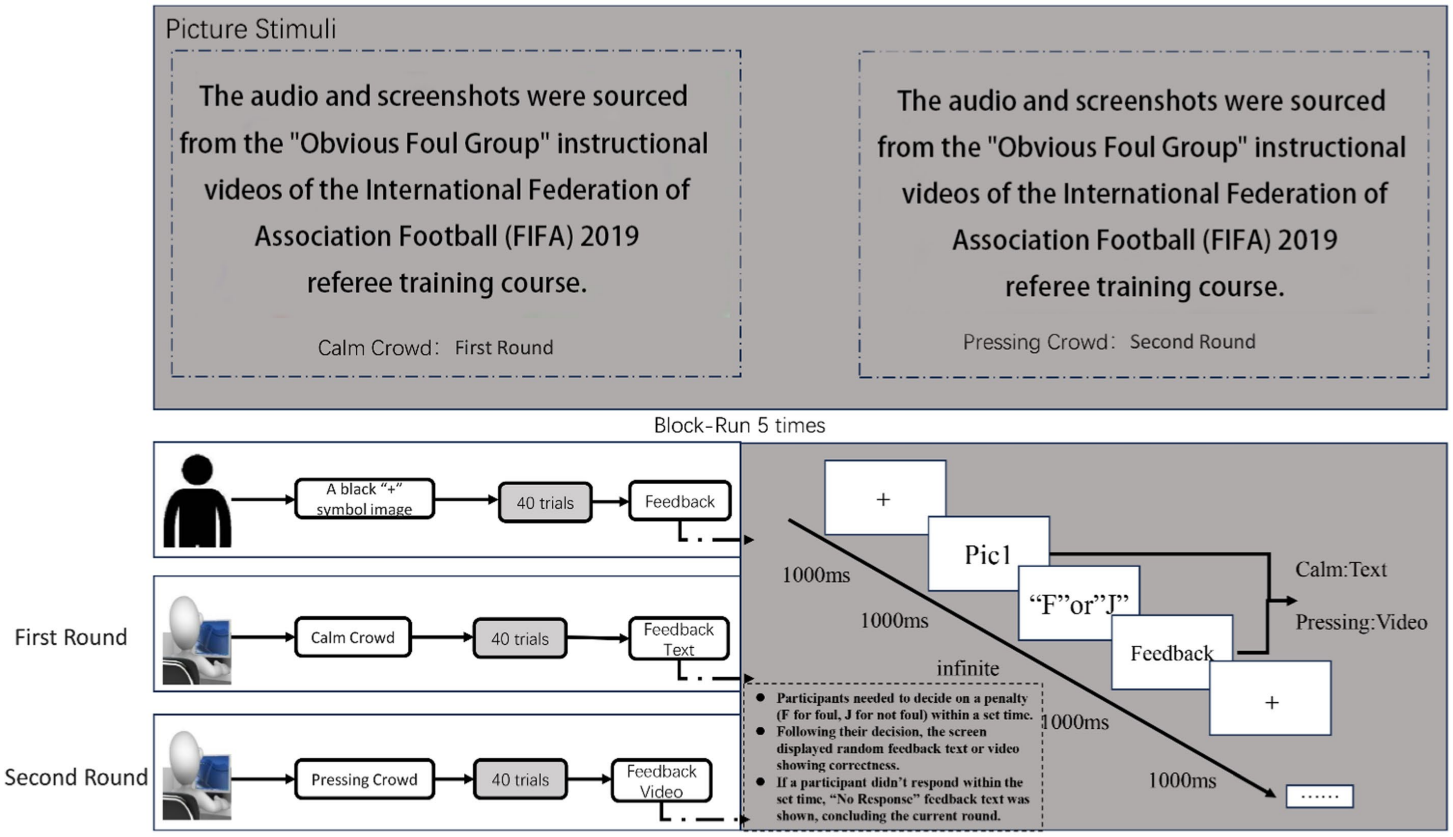
Procedure for the judgment decision task.

The study’s experimental design was grounded in the “information priming” effect paradigm. The experiment comprised two rounds, wherein visual priming information (specific soccer match images) was initially shown, followed by participants being asked to make penalty decisions. The Feedback was given to participants based on their decision outcomes. In the pressure-free environment of the first round, participants received image-based feedback. The second round simulated the pressure of an actual soccer match, with feedback presented as videos accompanied by sound. The initial experiment involved 200 test pairs. Each of the 40 soccer foul images was repeated 5 times, totaling 200 trials (40 × 5 = 200). During each test, participants first saw a “+” symbol image (for 800 milliseconds), then a soccer foul image. They needed to decide on a penalty (F for foul, J for not foul) within a set time. Following their decision, the screen displayed random feedback text showing correctness. If a participant did not respond within the set time, “No Response” feedback text was shown, concluding the current round. The detailed experimental procedure was as follows: a black “+” symbol fixation point appeared for 1,000 milliseconds, followed by a stimulus image shown for another 1,000 milliseconds. The feedback video was shown for 1,000 milliseconds. The interval between stimulus end and the next test round start ranged from 300 to 700 milliseconds, averaging 500 milliseconds. Following a rest period, participants started the second round of the experiment, mirroring the first. The distinction was that, for more authentic feedback, participants were shown videos capturing live audience reactions of “boos” and “cheers” after their decisions in real matches. When a participant did not respond within the set time, images showing “No Response” were displayed as feedback. The procedure for the judgment decision task is shown in Figure 2.

### 2.3 The data processing and analysis

The study utilized a Neuroscan 64-channel EEG recording system and Scan4.5 hardware and software system by Neuroscan to simultaneously record and analyze both EEG and behavioral data. The sampling frequency was set at 1,000 Hz, and at the commencement of the formal experiment, electrode impedance was reduced to below 5 kΩ. Video and audio materials were edited using iMovie and Adobe Audition 3.0 software, respectively. The testing of participants was conducted using a Dell XPS 15 laptop equipped with a 15.6-inch LED display screen. Bose QuietComfort 35 II over-ear head-phones were employed for auditory stimuli delivery. The experiment programming was carried out using E-prime Professional 2.0 software. Statistical analyses between groups of participants for both experiments were performed using SPSS 23.0 software.

**Figure 3:**
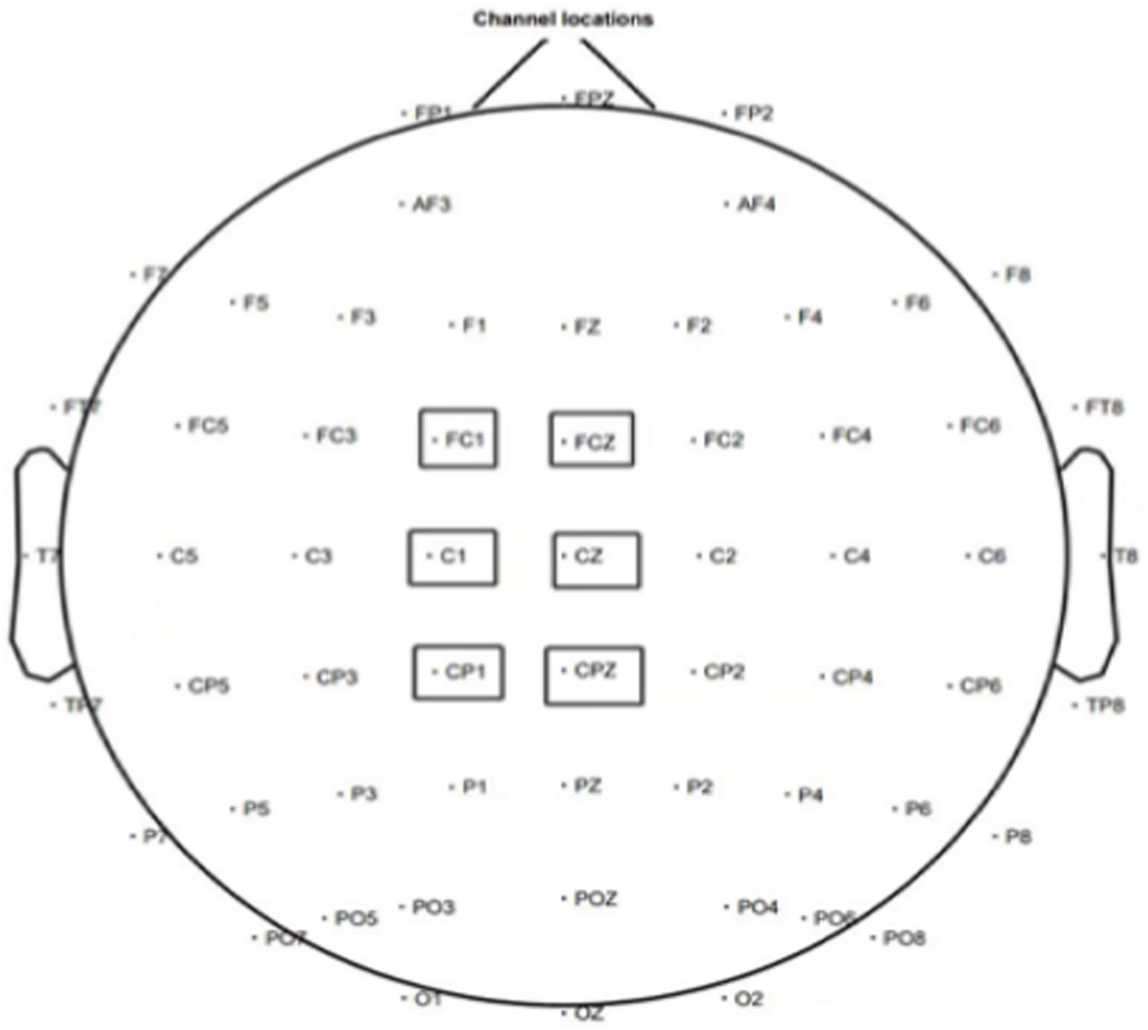
Overall distribution of 64 electrodes and selection of analytical electrodes.

We employed MATLAB for preprocessing the collected ERP data. Initially, we acquired electrode potential data with bilateral mastoid references (M1, M2). Secondly, we removed unnecessary electrodes, such as EEG, CB1, and CB2, and applied filtering operations (high-pass filter: 0.1 Hz, low-pass filter: 30 Hz). We also eliminated power line interference in the 48-52 Hz and 98–102 Hz range. Next, we segmented the data from 150 ms before to 500 ms after the decision. We also calibrated the baseline using data from 150 ms before point 0. This operation helps ensure that the observed changes in brain activity are more accurately attributed to the events of the study, rather than other unrelated fluctuations. Baseline calibration can reduce signal fluctuations caused by electrode shifts, environmental noise, etc., improving the signal-to-noise ratio of the data, thereby making the results more reliable. In event-related potential (ERP) studies, accurate time-locking and baseline calibration help identify and analyze specific ERP components related to cognitive processes, such as P300 or N400. This standardized processing not only improves the accuracy and reliability of research results but also ensures the reproducibility and comparability between different studies. After segmentation, we manually interpolated the problematic electrodes and removed the defective segments. Additionally, we employed ICA to remove artifacts. Finally, we conducted tests and eliminated ERP artifact fragments related to blinks, eye movements, EMG, and amplitudes exceeding ±80 μV. Following preprocessing, we superimposed and averaged all segmented participant data. We also performed superimposition and averaging of data among participants within the same group. By analyzing the obtained waveform, we observed significant P300 and N400 activity. They were determined based on the brain map and existing literature, with specific points being FC1, FCZ, C1, CZ, CP1, and CPZ, as depicted in Figure 3.

## 3 Results

### 3.1 Analysis of behavioral data results

**Figure 4:**
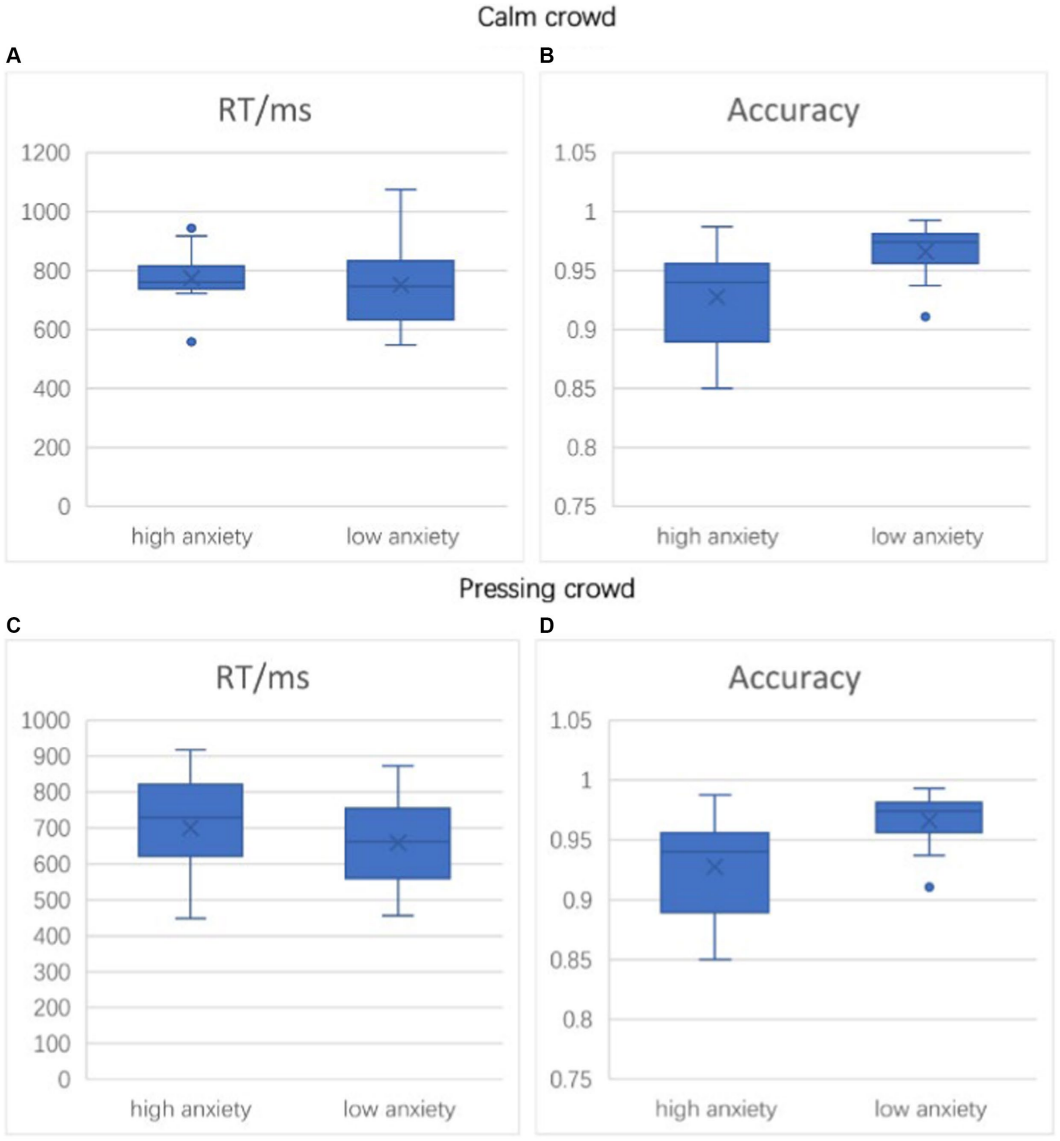
Box plot of the mean distribution of accuracy and response time between different conditions. **(A–D)** represent accuracy and reaction time under different conditions.

The experimental findings reveal that anxiety has a notable impact on the accuracy of penalty decisions made by decision-makers. In situations without pressure, the accuracy of decisions made by participants with high anxiety levels is notably inferior to that of participants with low anxiety levels (*F* = 3.709, *p* = 0.011 < 0.05). However, their response times exhibit no substantial distinction (*F* = 0.487, *p* = 0.109 > 0.05). In pressured environments, both the high-anxiety and low-anxiety groups show diminished response times compared to the no-pressure condition. In pressured situations, no significant difference in decision accuracy was observed among participants with varying levels of anxiety (*F* = 0.523, *p* = 0.115 > 0.05), indicating our study did not find sufficient evidence to support the hypothesis that anxiety levels affect decision accuracy under pressure. Detailed accuracy and response time data are presented in the Figure 4 (Tables 1, 2).

**Table 1:** The accuracy and reaction time of participants in Experiment 1.

Category	Pressing crowd (*n* = 34)		Calm crowd (*n* = 36)	
	Mean	Standard deviation	Mean	Standard deviation
Accuracy/%	92.75	0.040	96.60	0.020
Reaction time/ms	773.684	95.490	751.443	136.885

**Table 2:** The accuracy and reaction time of participants in Experiment 2.

Category	Pressing crowd (*n* = 34)		Calm crowd (*n* = 36)	
	Mean	Standard deviation	Mean	Standard deviation
Accuracy/%	96.60	0.016	96.73	0.021
Reaction time/ms	701.175	144.129	651.592	111.359

Previous researches indicate that anxiety might impact decision-makers’ accuracy in decision-making. Anxiety could result in inadequate attention concentration, reduced information processing capacity, and undue emphasis on risks, consequently affecting decision quality and accuracy. In addition, a moderate stress environment may affect the decision-making speed of athletes and referees. Especially when facing evaluative decisions related to penalty decisions, stress can shorten the reaction time of athletes and referees when performing tasks, which may be related to factors such as the need for rapid response, psychological activation, attention regulation, and automated response caused by stress (Baumeister, 1991). The behavioral data from this study further validate the previously mentioned perspectives. Specifically, elevated anxiety levels can impact decision-makers’ accuracy, while well-calibrated pressure can genuinely boost decision-makers’ decision speed. Furthermore, this study uncovers that suitable pressure can mitigate the adverse influence of anxiety on decision-makers’ decision-making capability. This is evidenced by the observation that no significant distinction in decision accuracy exists between participants of varying anxiety levels under pressure circumstances.

### 3.2 Analysis of EEG data results

#### 3.2.1 P300

**Figure 5:**
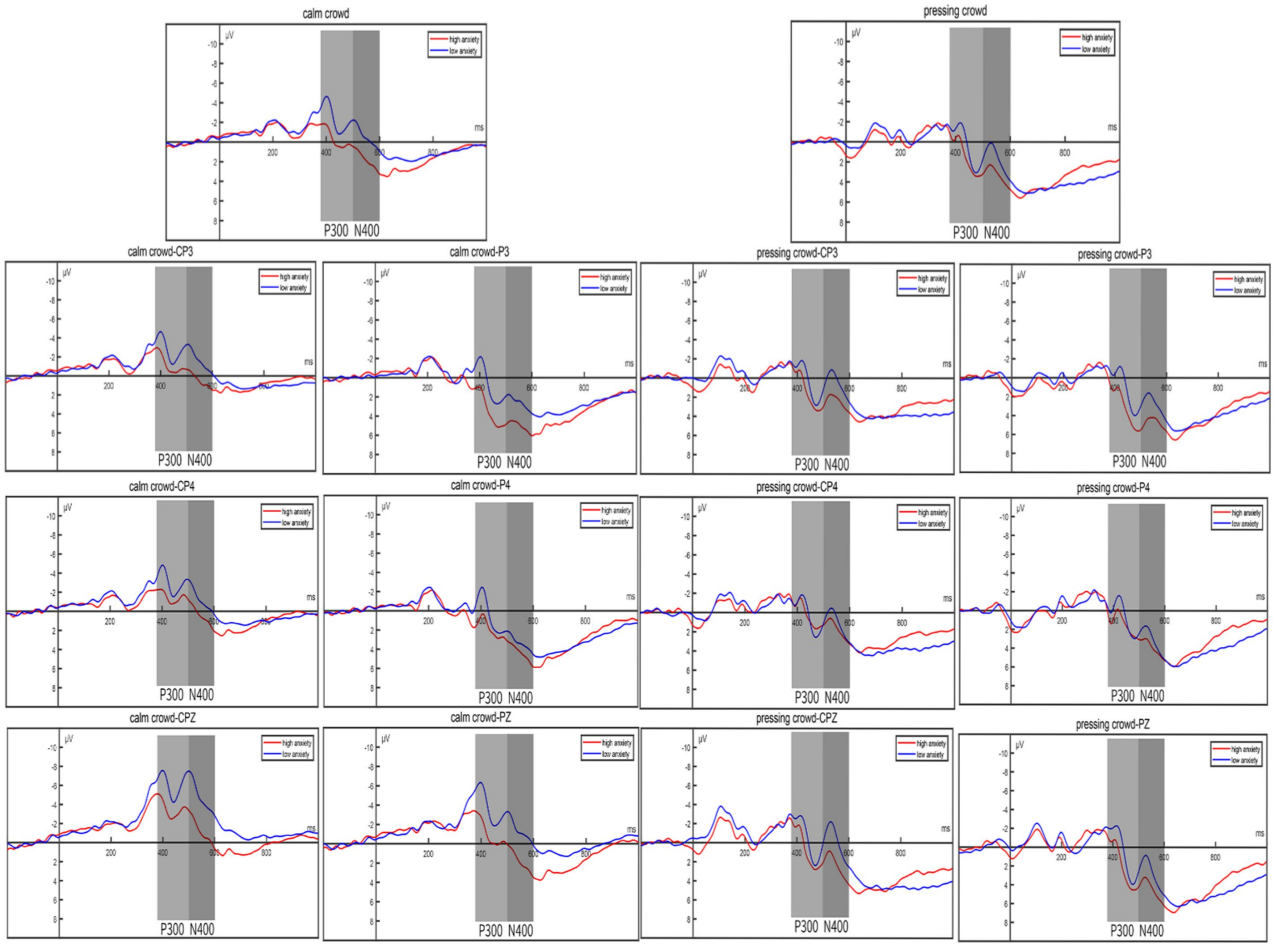
Event-related potential (ERP) results. Participants with different levels of anxiety under different pressure conditions in the average amplitude maps of P300 and N400 at 6 electrode points.

**Figure 6:**
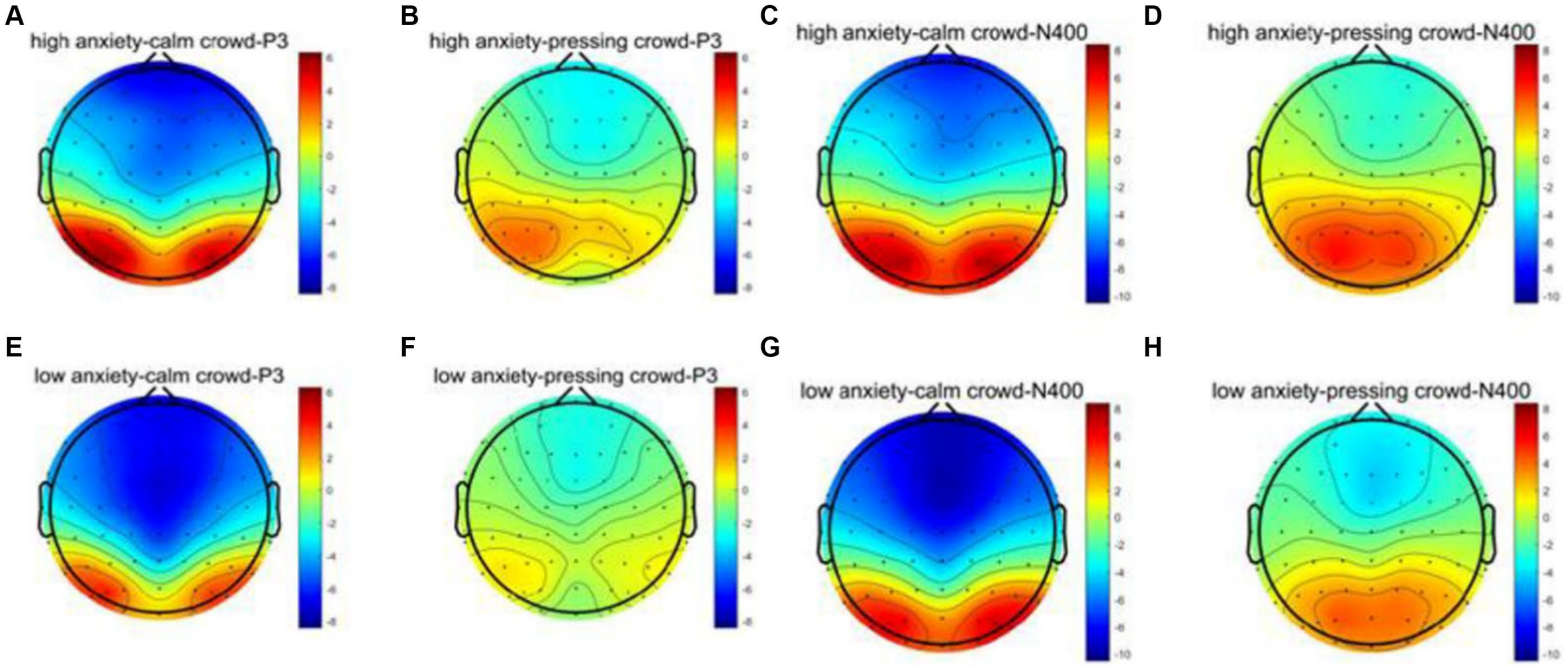
The topographic maps of P300 and N400 for different conditions. **(A–H)** represent the brain region evidence for the components P3 and N400 under various conditions.

According to relevant literature regarding the selection of P300 component sites, this study computed the average amplitude within the 380–480 ms range for six electrode sites: CP3, CPZ, CP4, P3, PZ, and P4. Subsequently, paired-sample *t*-tests were conducted for participants under both stress and non-stress conditions. The findings indicated a significant difference in P300 amplitudes between the high anxiety group and the low anxiety group under non-stress conditions. Specifically, the amplitude in the high anxiety group significantly increased (*p* = 0.0181, *t* = 2.4874). However, under stress conditions, no significant difference was observed in P300 amplitudes between the high anxiety and low anxiety groups (*p* = 0.2292, *t* = 1.2205). The waveform graph is presented in the following Figure 5, and the topographic maps in the following Figure 6.

#### 3.2.2 N400

Based on relevant literature regarding the selection of N400 component electrodes, this study computed the average amplitude from F3, FZ, F4, FC3, FCZ, and FC4. The average amplitude within the 480–600 ms time window was extracted. Separate independent samples *t*-tests were performed on the two groups of participants, one under no-pressure conditions and the other under pressure conditions. Ultimately, the study found significant differences in N400 amplitude between the high anxiety group and the low anxiety group under no-pressure conditions. The amplitude was notably higher in the high anxiety group (*p* = 0.0059, *t* = 2.9437). However, under pressure conditions, there was no significant difference in N400 amplitude between the high anxiety group and the low anxiety group (*p* = 0.1890, *t* = 1.3411). The waveform graph is shown in the following Figure 5, and the topographic maps in the following Figure 6.

## 4 Discussion

In real-life scenarios, the penalty decisions of soccer referees are frequently impacted by feelings of anxiety, and the pervasive pressure in soccer matches exacerbates this impact. This study devised an experiment simulating penalty decision-making scenario for referees in actual soccer matches. Participants were mandated to swiftly judge if a specific action amounted to a foul, followed by relevant feedback contingent on their determinations. The experimental outcomes demonstrate that referees with elevated anxiety levels evoke greater P300 and N400 amplitudes in an anxiety-free setting. Nevertheless, under pressured conditions, no notable distinctions in P300 and N400 amplitudes were observed between referees exhibiting high and low anxiety levels.

### 4.1 P300

The P300 is a distinctive event-related potential (ERP) component that emerges approximately between 300 and 1,000 milliseconds after stimulus presentation. Its manifestation is connected to the process of selecting actions during decision-making, influencing whether a decision is made. Simultaneously, researchers emphasize that the amplitude of the P300 is associated with the decision-making resources utilized by participants. As the consumption of decision resources intensifies during the decision-making process, the amplitude of the elicited P300 increases. Conversely, the amplitude of the P300 diminishes (Bishop, 2007). In this study, when external pressure was absent, referees with high anxiety levels exhibited notably greater P300 amplitudes in comparison to referees with low anxiety levels. This outcome substantiates the preceding hypothesis: anxiety emotions impact the decisions of soccer referees, resulting in heightened consumption of decision resources. The processing efficiency theory posits that in real-world decision-making, an individual’s brain resources, responsible for storage and processing, are engaged in handling emotions (such as anxiety and depression) and bodily sensations. Consequently, this diverts attention from immediate tasks. This reduced processing efficiency in the brain leads to a heightened allocation of resources to the ongoing task, ensuring its accomplishment (Polich, 2007; Sun and Zhang, 2013). Previous studies have demonstrated that individuals with elevated anxiety levels display heightened activation in brain regions such as the amygdala, anterior cingulate cortex, and orbitofrontal cortex while engaged in decision-making tasks. This implies that when affected by anxiety emotions, individuals need to allocate increased decision resources to complete tasks requiring immediate decisions (Hardy and Hutchinson, 2007). Within this experiment, referees of different anxiety levels confronted equal task complexities in making penalty decisions. Nonetheless, those with elevated anxiety levels, influenced by their anxious condition, required additional decision resources for penalty decisions, leading to an augmented P300 amplitude. When pressure conditions were present, the amplitude of the P300 elicited from referees with high anxiety levels did not exhibit a notable difference compared to those with low anxiety levels. Along with findings from pressure-free conditions, this implies that pressure elements mitigate the influence of anxiety on referees’ penalty decision-making. As per the Yerkes-Dodson Law, pressure stimulates responses in the sympathetic nervous system and prompts the release of stress hormones like cortisol, mobilizing bodily resources to effectively manage challenges (Koehn, 2013). Extensive inter-views with referees and athletes have previously revealed that seasoned athletes and referees frequently hold the capability to adapt constructively to pressure. Moderate pressure can indeed enhance their concentration, resulting in a state akin to “flow,” ultimately enhancing their performance (Diamond et al., 2007). Sapolsky emphasizes that the pivotal aspect in the Yerkes-Dodson Law lies in the appropriateness of pressure. Its impact is advantageous under conditions of mild to moderate pressure (on the left side of the inverted-U curve), but severe pressure results in an opposing effect (Koehn, 2013). In simpler terms, intense pressure is harmful to individuals, whereas mild pressure is not, especially when individuals feel safe in the pressure situation (such as voluntarily riding a roller coaster). This interpretation also elucidates the outcomes of the conducted experiment. Given the inherently calm and appropriate laboratory setting, and considering that the experiment’s pressure stimulus originates from chosen videos featuring non-aggressive audience responses, the pressure variable in this study remains confined to a logical extent. Furthermore, the participants, all football referees with relevant refereeing experience, have the competency and familiarity to adapt to pressure circumstances. Thus, in this study, the pressure element effectively assists referees with elevated anxiety levels to enhance their attentional focus, allocate increased cognitive resources to decision-making tasks, lower the complexity of the decision task, and consequently decrease the utilization of decision resources. This outcome is evident in the experiment, where there exists no noteworthy disparity in P300 amplitude when compared to the low anxiety group.

### 4.2 N400

The N400 is a negative waveform usually detected at central and top electrode locations, frequently associated with breaches in semantic expectancy and the retrieval of stimulus-related information. It is widely believed by researchers to reflect the neural activity of individuals seeking and activating specific semantic or stimulus-related details (Sapolsky, 2015). The N400’s amplitude increases with participants’ greater effort in retrieving stimulus-related knowledge; conversely, with lesser effort, the amplitude decreases (Lau et al., 2008). In this study, referees experiencing external pressure and high anxiety levels showed notably larger N400 amplitudes than those with low anxiety levels. This is consistent with the earlier hypothesis of this study: anxious emotions impair referees’ capacity to access relevant working memory and integration skills, thus influencing penalty decision-making. Existing neurocognitive research has extensively showcased that anxiety emotions adversely impact participants’ performance in cognitive processing tasks. Furthermore, prior neurocognitive research has well evidenced the detrimental influence of anxiety emotions on participants’ cognitive processing task performance. As pointed out by Sapolsky, a crucial con-sideration in the Yerkes-Dodson law is the appropriateness of pressure. The impact of mild to moderate pressure (to the left of the U) is advantageous, while intense pressure leads to an opposite outcome (Kutas and Federmeier, 2011). Moreover, this study establishes that the N400 is a pivotal measure signifying the processing efficiency of a cohort of soccer referees in penalty decision tasks. It also forms the basis for future investigations into the interplay of factors like pressure, experience, and self-confidence that impact individual task decision efficiency.

Conversely, similar to the P300, the amplitude of the N400 induced in referees with high anxiety levels does not significantly deviate from that in referees with low anxiety levels when subjected to pressure. The underlying reason could be analogous: as per the “Yerkes-Dodson Law,” pressure’s influence on individual decision-making often follows an inverted-U pattern, suggesting that moderate pressure frequently enhances decision-makers’ capacity (Sapolsky, 2015). Meanwhile, this study also establishes the N400’s role as a pivotal metric signifying the processing efficiency of a group of soccer referees in penalty decision tasks. This study simultaneously paves the way for future investigations into the mechanisms behind the influence of factors like pressure, experience, and self-confidence on individual task decision efficiency, and their interplay.

## 5 Conclusion

This study utilizes established theories such as cognitive resource theory, processing efficiency theory, and the Yerkes-Dodson law to examine how neural variations manifest in penalty decision-making among soccer referees with different anxiety levels within a simulated real-game pressure scenario. Using neuroscientific research techniques, we investigated the neural processes influencing penalty decision-making in referees with varying anxiety levels under pressure. The results show that during tasks of equal difficulty without pressure, referees with higher anxiety levels demonstrate notably greater amplitudes of both P300 and N400 in comparison to referees with lower anxiety levels. This implies that anxiety predominantly affects referees’ penalty decision-making by diminishing their cognitive resources and decision efficiency. Yet, under pressured circumstances, no noteworthy distinctions emerged in the amplitudes of P300 and N400 induced in referees with varying anxiety levels. This implies that suitable pressure can alleviate the disruption caused by anxiety to cognitive resources and processing efficiency, enhancing decision-makers’ ability to focus on decision tasks. The study’s outcomes corroborate the notion that suitable pressure mitigates the influence of anxiety on referees’ penalty decision-making, in accordance with the viewpoints of researchers. Furthermore, this study partially offers a novel explanatory framework regarding the observed correlation between pressure and individual performance within the framework of the Yerkes-Dodson law. Compared to prior research, this study employs neuroscientific techniques to reveal the neural mechanisms by which anxiety impacts individual decision-making. It elucidates the foundational brain mechanisms driving this influence and clarifies the reasons for anxiety’s impact on referees’ penalty decisions, as well as the alterations in this impact under pressure. This study rectifies gaps in prior research concerning the mechanisms through which emotions influence penalty decision-making. It also provides a deeper comprehension of how emotions influence referees’ unbiased judgment in soccer matches, employing a neuroscientific standpoint. In the next phase of our research, we plan to adjust the feedback materials used in our experiments by employing video and audio materials that have similar perceptual loads. This adjustment is intended to minimize the baseline differences caused by various sensory stimuli. By optimizing the experimental procedures and enhancing our data interpretation capabilities, we aim to gain a more detailed understanding of how stress and anxiety jointly affect the behavior and decision-making processes of referees.

In future research, we aim to further consider factors such as officiating experience, gender, age, and cultural background that may influence soccer referees’ decision-making, integrating technologies like functional magnetic resonance imaging (fMRI) or near-infrared spectroscopy (NIRS) to uncover the brain regions and networks involved in referees’ decision-making processes. These findings are expected to foster interdisciplinary integration among sports science, neuroscience, and psychology, providing deeper scientific insights into the cognitive and emotional mechanisms within referees’ decision-making processes. Furthermore, this will enhance our understanding of cognitive behavioral patterns in high-pressure environments, particularly regarding how the brain coordinates and processes complex information during decision-making, thus demonstrating significant theoretical value and broad application prospects.

## Data availability statement

The raw data supporting the conclusions of this article will be made available by the authors, without undue reservation.

## Ethics statement

The studies involving humans were approved by Ethics Committee of Xi’an physical education university. The studies were conducted in accordance with the local legislation and institutional requirements. The participants provided their written informed consent to participate in this study.

## Author contributions

LZ: Conceptualization, Data curation, Formal analysis, Funding acquisition, Investigation, Methodology, Project administration, Resources, Software, Supervision, Validation, Visualization, Writing – original draft, Writing – review & editing. HS: Visualization, Writing – review & editing. HZ: Conceptualization, Data curation, Formal analysis, Funding acquisition, Investigation, Methodology, Project administration, Resources, Software, Supervision, Validation, Visualization, Writing – original draft, Writing – review & editing. JD: Conceptualization, Data curation, Formal analysis, Funding acquisition, Investigation, Methodology, Project administration, Resources, Software, Supervision, Validation, Visualization, Writing – original draft, Writing – review & editing. ZW: Visualization, Writing – review & editing.
